# A dual-path mediation model: associations between childhood psychological maltreatment and loneliness among Chinese college students

**DOI:** 10.3389/fpsyt.2026.1747337

**Published:** 2026-02-02

**Authors:** Zihan Zhou, Mingbo Liu, Liang Chen

**Affiliations:** 1 The School of Social Development and Public Policy, Fudan University, Shanghai, China; 2 Counseling and Psychological Services, Fudan University, Shanghai, China; 3 Shanghai Academy of Educational Sciences, Shanghai, China; 4 Research Center for Psychological Development, University of Science and Technology Liaoning, Anshan, Liaoning, China; 5 School of Marxism, University of Science and Technology Liaoning, Anshan, Liaoning, China

**Keywords:** childhood psychological maltreatment, college students, loneliness, positive coping styles, trust in others

## Abstract

**Objective:**

This study aimed to examine the potential psychological mechanisms that may link childhood psychological maltreatment to loneliness among Chinese college students by testing a dual-path mediation model involving trust in others and positive coping styles.

**Methods:**

A cross-sectional survey was conducted with 603 college students using self-report measures of childhood psychological maltreatment, trust in others, positive coping styles, and loneliness. Data were analyzed using correlation analysis and structural equation modeling with bootstrapping.

**Results:**

Childhood psychological maltreatment was positively correlated with loneliness and negatively correlated with trust in others and positive coping. Trust in others and positive coping styles were negatively correlated with loneliness. The structural equation model revealed significant indirect effects: childhood maltreatment was associated with loneliness both through reduced trust in others (indirect effect = 0.063, 12.07% of total effect) and through weakened positive coping styles (indirect effect = 0.084, 16.19% of total effect). The total indirect effect accounted for 28.26% of the total variance.

**Conclusion:**

The findings support the proposed dual-path model, suggesting that the association between childhood psychological maltreatment and later loneliness may operate through eroded interpersonal trust and impaired development of adaptive coping strategies. This underscores the importance of integrated interventions targeting both trust reconstruction and coping skills training in mitigating the long-term impacts of early adversity.

## 1 Introduction

Loneliness is a negative emotional experience arising from the perceived gap between one’s social connections and their expectations in social interactions (1). In recent years, with the expansion of college enrollments, increased academic pressure, and intensified social competition, the issue of loneliness among college students has become increasingly prominent. According to “The Report on the Mental Health Status of Chinese College Students (2023)”, approximately 34.7% of college students experience varying degrees of loneliness, with 12.4% suffering from severe loneliness. Loneliness not only affects college students’ mental health but also exerts a profound impact on their academic performance, interpersonal relationships, self-identity, and future development. Academically, loneliness is significantly associated with academic burnout and decreased learning motivation (2). Lonely college students often lack a learning support system, struggle to cope with academic pressure effectively, leading to poor grades and an increased risk of dropping out. In terms of life adaptation, loneliness is closely linked to behavioral problems such as poor sleep quality, irregular eating, and internet addiction (3). Furthermore, long-term loneliness may trigger psychological disorders such as depression and anxiety, and even increase the risk of suicide (4). Therefore, in-depth exploration of the formation mechanism of college students’ loneliness and its intervention paths has become an important topic in college mental health education.

### 1.1 The relationship between childhood psychological maltreatment and college students’ loneliness

Childhood psychological maltreatment refers to persistent non-physical harmful behaviors by caregivers toward children, such as intimidation, belittlement, neglect, and interference (5). As a core form of child maltreatment, psychological maltreatment has long-term and hidden negative impacts on individual development. According to attachment theory (6), early rejection or neglect by caregivers disrupts children’s secure attachment patterns, leading them to form an internal working model (IWMs) of “others are untrustworthy.” This negative interpersonal schema persists into adulthood, causing difficulties in establishing and maintaining intimate relationships, thereby resulting in loneliness. Numerous studies have shown that childhood psychological maltreatment is an important distal risk factor for loneliness in adulthood (7–9). In addition, social information processing theory suggests that maltreated children are more likely to develop a hostile attribution bias, interpreting neutral social cues as threats and thus avoiding social interactions (10). Domestic studies have also found that experiences of childhood psychological maltreatment can significantly predict the level of loneliness among college students (11). Individuals who have experienced childhood maltreatment may withdraw from society; due to feelings of inferiority and pain, they are unwilling to contact others, thus failing to obtain social support or care, and may eventually become sensitive to loneliness (9, 12).

While the association between childhood psychological maltreatment and later loneliness is established, the specific psychological mechanisms transmitting this effect remain inadequately understood. Existing studies have often examined single mediators, such as emotion dysregulation (13) or maladaptive schemas (14), or have focused on broader negative outcomes like depression which is highly comorbid with loneliness (15). Few studies have integrated distinct interpersonal and intrapersonal pathways to specifically predict loneliness. Furthermore, the roles of generalized trust in others and positive coping styles as parallel mediators in this context have not been sufficiently explored within a unified model. To address these gaps, this study proposes and tests an integrative dual-path mediation model. This model posits that childhood psychological maltreatment is associated with loneliness through two potentially interrelated yet distinct mechanisms: (1) by eroding the fundamental capacity to trust others, and (2) by impairing the development of adaptive, positive coping strategies.

### 1.2 The mediating role of trust in others

Trust in others is a psychological state in which individuals are willing to take risks based on positive expectations of others’ goodwill and reliability (16). Contemporary developmental theory, particularly the concept of *epistemic trust*—the capacity to trust others as reliable sources of knowledge and social information—provides a nuanced framework for understanding this erosion (17, 18). Epistemic trust is posited to develop within secure attachment relationships where the child’s communicative intentions are recognized and validated. Chronic psychological maltreatment constitutes an “epistemic mistrust” environment, invalidating the child’s experiences and impairing their ability to learn from social interactions (17). This disruption leads children to form a generalized schema of others as untrustworthy (19) and adopt defensive alienation strategies (20). Empirical work links adverse childhood experiences to impaired epistemic trust, which in turn predicts social alienation and psychopathology, including features related to loneliness (21). Consistent with this, studies find childhood emotional maltreatment negatively predicts interpersonal trust in college students (22).

Trust in others is one of the influencing factors of loneliness. Studies have found that the higher the interpersonal trust score of college students, the lower their loneliness score, and social anxiety plays a partial mediating role between the two (23). A survey of 850 college students by Zhou and Yao (24) found that psychoticism and neuroticism not only directly predict loneliness but also have an indirect effect by reducing interpersonal trust, suggesting that trust is a key mediator in the influence of personality traits on loneliness. Since the college years are a critical period for individuals to establish new social relationships, a low level of trust will hinder their integration into peer groups, thereby exacerbating the experience of loneliness.

The proposed pathway through trust in others is grounded in Attachment Theory (6). Chronic psychological maltreatment by caregivers disrupts the formation of a secure base, leading children to develop negative internal working models (IWMs) characterized by the belief that “others are untrustworthy” and “the self is unlovable” (19). Recent mechanistic evidence strengthens the plausibility of such parallel pathways. In psychiatric outpatients, emotional abuse increases psychological distress serially through (a) metacognitive/emotional beliefs and (b) repetitive negative thinking (25, PMIP)—processes conceptually akin to the trust and coping mechanisms proposed here. Likewise, college-student studies show that childhood trauma predicts depression via repetitive negative thinking and experiential avoidance (15), while early maladaptive schemas simultaneously erode interpersonal trust (disconnection/rejection domain) and coping resources (impaired-autonomy domain; 14). Furthermore, from a Social Information Processing perspective, individuals with such IWMs are prone to a hostile attribution bias, interpreting ambiguous social cues as threatening or rejecting (10). This cognitive bias fosters vigilance and social withdrawal, effectively reducing opportunities for positive social engagement and reinforcing feelings of loneliness. An invalidating family environment, central to psychological maltreatment, has been shown to critically shape such socio-cognitive processes and subsequent psychopathology (13).

Taken together, two empirical gaps justify the present study. First, although trust and coping have been examined separately as correlates of loneliness, their simultaneous, theory-driven mediation has not been tested. Second, no study has situated this dual process within collectivistic university contexts where reciprocal “guanxi” networks may amplify the costs of low trust and ineffective coping. We therefore constructed a dual-path model that positions (a) trust in others and (b) positive coping styles as parallel mediators linking recalled childhood psychological maltreatment to current loneliness.

### 1.3 The mediating role of positive coping styles

The second pathway is explained through Conservation of Resources Theory (26). Childhood psychological maltreatment is conceptualized as a chronic and severe loss of psychological resources. It depletes a child’s pool of vital resources such as self-esteem, sense of control, and security. This resource loss impedes the development of robust internal assets (e.g., self-efficacy, optimism) necessary to effectively engage with and manage subsequent life stressors, including the social challenges of college life. Consequently, maltreated individuals often enter adulthood with a deficit in adaptive coping repertoires. This is consistent with research showing that childhood trauma predicts greater use of maladaptive strategies like experiential avoidance and repetitive negative thinking (14, 15), which are antithetical to positive coping. Without the resources to deploy positive coping strategies (e.g., active problem-solving, seeking support, positive reappraisal), individuals are more likely to feel overwhelmed and helpless when faced with difficulties, thereby increasing their vulnerability to loneliness.

Coping styles refer to individuals’ cognitive and behavioral response patterns when facing stressful events (27). Positive coping (such as seeking support and problem-solving) can effectively alleviate the negative impacts of stress, while negative coping (such as avoidance and self-blame) may exacerbate psychological distress. Childhood psychological maltreatment impairs individuals’ coping resources: maltreated children often lack effective emotion regulation strategies and tend to adopt negative coping styles rather than positive ones (28). Therefore, childhood psychological maltreatment predicts insufficient coping skills in individuals, making it more difficult for maltreated children to face and handle stress.

Compared with non-maltreated children, individuals who have experienced childhood psychological maltreatment use fewer coping skills, and low coping skills may exacerbate depressive symptoms (which are highly covariant with loneliness). A study by Li et al. (29) pointed out that positive coping plays a significant mediating role between childhood psychological maltreatment and depression in college students. In addition, a study by Xie et al. (30) found that positive coping reduces the experience of loneliness by enhancing the perception of social support. Therefore, childhood psychological maltreatment may inhibit the development of positive coping strategies, making individuals more likely to fall into a state of helplessness when facing social challenges in college life, thereby resulting in loneliness.

Drawing on the integrated theoretical framework outlined above, this study tests a dual-path mediation model. We hypothesize that childhood psychological maltreatment is associated with elevated loneliness in college students through two distinct mediating pathways:

Hypothesis 1: Trust in others mediates the relationship between childhood psychological maltreatment and loneliness. Specifically, childhood psychological maltreatment is predicted to erode generalized trust in others, which in turn is linked to increased feelings of loneliness.
Hypothesis 2: Positive coping styles mediate the relationship between childhood psychological maltreatment and loneliness. Specifically, childhood psychological maltreatment is associated with hinder the development of positive coping styles, and this deficiency in adaptive coping is subsequently associated with greater loneliness.

## 2 Methods

### 2.1 Participants

This survey adopted the principle of stratified cluster sampling, with classes as the unit, and tests were conducted during self-study time, with questionnaires collected on the spot. College students from two universities in Liaoning and Shanghai were selected as the subjects. All participants signed informed consent forms. A total of 668 questionnaires were distributed, 653 were received, and 40 invalid questionnaires were excluded, resulting in a final valid sample of 603. Among them, 396 were male (65.7%) and 207 were female (34.3%). The number of students with urban household registration was 335, accounting for 55.6%; those with rural household registration was 268, accounting for 44.4%. The age range of the participants was 18–22 years, with an average age of 19.70 years and a standard deviation of 1.14.

### 2.2 Measures

#### 2.2.1 Childhood psychological abuse scale

Childhood Psychological Abuse Scale compiled by Pan et al. (31) was used. This scale includes 5 subscales: intimidation, neglect, belittlement, interference, and indulgence, with a total of 23 items. It uses a 5-point Likert scale, with scores ranging from 0 (never) to 4 (always). A higher total score indicates a higher degree of childhood psychological maltreatment perceived by the participants. In this study, the internal consistency reliability coefficients of the 5 subscales (intimidation, neglect, belittlement, interference, and indulgence) were 0.920, 0.871, 0.912, 0.833, and 0.601, respectively.

#### 2.2.2 Simplified coping style questionnaire

Simplified Coping Style Questionnaire compiled by Xie (32) was used. This scale includes 2 subscales: positive coping style and negative coping style, with 12 items in the former and 8 items in the latter, totaling 20 items. All items use a 4-point Likert scale (1 = never used, 4 = often used), and a higher score indicates that the individual uses the coping style more frequently. In this study, the internal consistency reliability coefficient of the positive coping style subscale was 0.932.

#### 2.2.3 Faith in people scale

Faith in People Scale compiled by Rosenberg (33) was used. The Chinese version of the scale is used to assess whether the subjects have confidence in the trustworthiness, honesty, kindness, generosity, and friendliness of people in general (34). This scale includes 2 optional items and 3 agree-disagree items, all using a 2-point scoring system. Positive answers indicate that the subjects lack general confidence in human nature. The total score of the scale ranges from 1 (confidence in all five items) to 6 (no confidence in all five items). The internal consistency reliability coefficient of the questionnaire in this study was 0.78.

#### 2.2.4 UCLA loneliness scale

UCLA Loneliness Scale was compiled by Russell et al. (35). The Chinese version of the loneliness scale contains 20 items (34), using a 4-point Likert scale, with scores ranging from 1 (never) to 4 (always), all scored positively. A higher score indicates stronger loneliness. In this study, the internal consistency reliability coefficient of the loneliness scale was 0.914.

### 2.3 Data analysis

SPSS 22.0 and Mplus 8.1 were used for data management and analysis, including reliability analysis, correlation analysis, factor analysis, and structural equation modeling.

## 3 Results

### 3.1 Common method bias test

To avoid common method biases in the analysis of self-reported questionnaires, all questionnaires were filled out anonymously to improve the authenticity of the participants’ responses. Harman’s single-factor test was used to conduct exploratory factor analysis on 60 items of the 4 scales, and a total of 8 factors with eigenvalues greater than 1 were extracted, with the first factor explaining 28.992% of the variance, which is less than the critical standard of 40%. Therefore, no common method bias was detected in this study.

### 3.2 Correlation analysis between variables

The results showed that there were significant correlations between childhood psychological maltreatment, trust in others, positive coping styles, and loneliness (see **Table 1**). Specifically, childhood psychological maltreatment was significantly positively correlated with loneliness, and significantly negatively correlated with trust in others and positive coping styles. Trust in others and positive coping styles were significantly negatively correlated with loneliness.

**Table 1:** Descriptive statistics and correlation matrix for each variable (*N* = 603).

Variable	*M*	*SD*	1	2	3	4
1 CPA	0.74	0.68	1			
2 Trust others	4.36	1.40	-0.190^**^	1		
3 Positive coping style	2.89	0.64	-0.188^**^	0.232^**^	1	
4 Loneliness	2.03	0.53	0.461^**^	-0.458^**^	-0.454^**^	1

^**^p < 0.01. CPA, Childhood psychological abuse.

### 3.3 Mediation effect analysis

Since childhood psychological maltreatment, trust in others, positive coping styles, and loneliness are all latent variables, it is necessary to establish a structural equation model. Among them, the observed variables of trust in others are the scale items, and the observed variables of childhood psychological maltreatment are composed of subscales. According to the parceling theory, positive coping styles and loneliness use item parcels as observed variables. The bias-corrected nonparametric percentile Bootstrap estimation method was used for testing, and all variables were standardized. First, the total effect model of childhood psychological maltreatment on loneliness was established to test the total effect c and its significance. The results showed that the total effect of childhood psychological maltreatment on loneliness was 0.524, and the total effect coefficient was significant (p < 0.001), with overall good fitting indices (see **Table 2**).

**Table 2:** Total effect model and mediating model fit indices.

Model	*χ²*	*df*	CFI	TLI	SRMR	RMSEA
Total effect model	46.981	19	0.993	0.989	0.019	0.049
Mediating model	351.987	99	0.956	0.947	0.072	0.065

**Figure 1:**
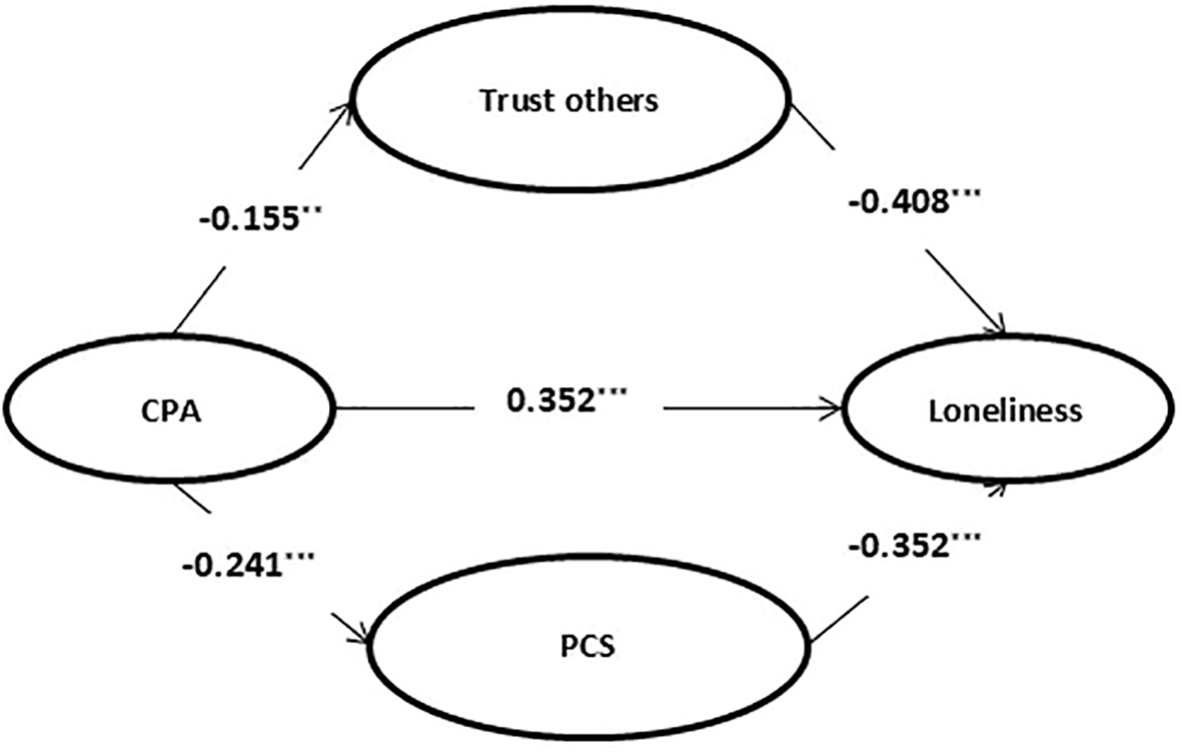
Mediating model of the relationship between childhood psychological maltreatment and loneliness. ^**^p < 0.01, ^***^p < 0.001. CPA, Childhood psychological abuse; PCS, Positive coping style.

Second, the significance of path coefficients was tested sequentially. In this study, a mediation model was established with childhood psychological maltreatment as the independent variable, loneliness as the dependent variable, and trust in others and positive coping styles as mediating variables (see **Figure 1**). The structural equation model analysis showed that all fitting indices were good (see **Table 2**), indicating that the model was relatively standard. Therefore, trust in others and positive coping styles play a partial mediating role in the relationship between childhood psychological maltreatment and loneliness, and this mediating role includes two paths: the separate mediating role of trust in others and the separate mediating role of positive coping styles.

Finally, the 95% confidence interval of the path coefficients was estimated with 1000 repeated samplings. The results showed that the mediating effect included two indirect effects: first, the standardized indirect effect 1 generated by the path of childhood psychological maltreatment → trust in others → loneliness, with a 95% confidence interval of (0.022, 0.111), which does not contain 0, and the mediating effect was 0.063, accounting for 12.07% of the total effect; second, the standardized indirect effect 2 generated by the path of childhood psychological maltreatment → positive coping styles → loneliness, with a 95% confidence interval of (0.038, 0.092), which does not contain 0, and the mediating effect was 0.084, accounting for 16.19% of the total effect. Therefore, the total mediating effect was 28.26%.

## 4 Discussion

### 4.1 The relationship between childhood psychological maltreatment and college students’ loneliness

This study found that childhood psychological maltreatment has a significant positive predictive effect on college students’ loneliness, which is consistent with previous research results (8, 36), suggesting that childhood psychological maltreatment is a relatively independent and stable distal risk factor for college students’ loneliness. It is important to note that while the present study did not collect neurobiological data, existing literature suggests that early chronic psychological maltreatment may lead to overactivation of the HPA axis and functional imbalance in the amygdala–prefrontal circuit (37). Such neurobiological sensitization might render individuals abnormally sensitive to social evaluation, thereby increasing the likelihood of social avoidance and loneliness (38). However, these mechanisms remain speculative in the context of our correlational data and warrant direct examination through neuroscientific methods in future research.

Attachment theory provides a core explanatory framework for this “time extension effect.” Children who repeatedly experience intimidation, belittlement, or emotional neglect find it difficult to establish secure attachments with their primary caregivers, thereby forming an internal working model (IWMs) of “others are untrustworthy/oneself is unlovable.” When entering college, individuals face a new social ecosystem (dormitories, clubs, romantic relationships) and need to re-establish interpersonal networks. If the internal working model is not corrected, individuals tend to interpret others’ intentions in a hostile or avoidant manner, leading to increased social withdrawal and loneliness (39). Dodge’s social information processing model further points out that maltreated children may have biases in the four stages of “cue encoding - interpretation - goal setting - response decision”: they are more likely to notice and amplify the threatening components in social cues, interpret neutral behaviors as rejection, and then choose avoidance or aggressive responses. This bias reduces the opportunities for positive interactions, forming a vicious cycle of “low interaction - high loneliness.”

### 4.2 The mediating role of trust in others

The structural equation model showed that trust in others plays a partial mediating role between childhood psychological maltreatment and college students’ loneliness. This indicates that childhood psychological maltreatment not only directly increases loneliness but also indirectly exacerbates the experience of loneliness by weakening the belief in others’ reliability and goodwill, which is consistent with previous research conclusions on the relationship between childhood trauma, interpersonal trust, and loneliness (e.g., 40, 41). The observed pathway through trust aligns with contemporary extensions of attachment theory focusing on epistemic trust (18). Psychological maltreatment, characterized by emotional invalidation and inconsistent responsiveness, can critically impair the development of epistemic trust—the conviction that social and emotional information from others is genuine, relevant, and applicable to the self (17). Individuals with compromised epistemic trust may become “closed off” to social learning, dismissing positive social cues or support, which maintains social wariness and isolation, thereby fueling loneliness (21). This mechanism offers a refined explanation for how early negative internal working models perpetuate social difficulties into adulthood. According to attachment theory (6), the relationship between maltreated children and their caregivers is often unstable, unreliable, and even harmful. Long-term psychological maltreatment behaviors such as devaluation and neglect from parents make it difficult for children to establish basic trust in others. A Chinese study on 214 college students found that interpersonal trust was significantly negatively correlated with emotional neglect and emotional abuse in childhood trauma experiences, and emotional abuse and emotional neglect in childhood trauma could negatively predict interpersonal trust (42). Therefore, psychological maltreatment undermines children’s trust in others, making them tend to remain vigilant and difficult to trust others even when facing new interpersonal relationships in adulthood.

According to social exchange theory (43), good interpersonal relationships are based on mutual trust, support, and reciprocity. When individuals have low trust in others, they will reduce their investment in interpersonal interactions and find it difficult to establish deep emotional connections with others. The lack of trust makes it difficult for college students to obtain emotional support and companionship in interpersonal interactions, failing to meet their needs for social relationships, thus leading to loneliness. In addition, the “attachment-interpersonal cognition-emotional outcome” cascade model proposed by Collins et al. (44) is a dynamic framework integrating attachment theory, social cognitive theory, and emotional regulation mechanisms. This model reveals how individuals’ early attachment experiences ultimately affect their emotional health and social adaptation by shaping interpersonal cognitive patterns. The core path of the model is: attachment pattern → interpersonal cognition → emotional outcome, where interpersonal cognition is a key mediating variable connecting early experiences and emotional adaptation. The results of this study also support this path: psychological maltreatment significantly negatively predicts trust in others, and trust in others significantly negatively predicts loneliness. In cultural contexts such as China’s, which emphasize collectivism, interpersonal trust and social exchange are often deeply embedded within networks of “guanxi” (relationships) and reciprocal obligations. Maltreatment experiences may make individuals deeply skeptical of such relational foundations, potentially creating a mismatch with the college social ecosystem that often operates on similar principles. Studies have shown that students with high loneliness often report “fear of owing favors” and “worry about being used,” a negative interpretation of reciprocal norms that may further weaken the foundation of trust. Although the current study did not include cross-cultural comparisons, this interpretation draws upon prior research conducted in Chinese cultural contexts (e.g., 42). Future studies should test the generalizability of these culturally nuanced pathways across diverse cultural settings.

This finding underscores the sequence from early adverse attachment experiences (maltreatment) to the formation of negative interpersonal cognitions (low trust), and finally to negative emotional outcomes (loneliness), as articulated by the cascade model and consistent with theories linking invalidating environments to socio-emotional deficits (13).

### 4.3 The mediating role of positive coping styles

Positive coping styles also show a significant partial mediating effect. The specific path is: psychological maltreatment → positive coping → loneliness. A large number of studies have shown that individuals who have experienced childhood psychological maltreatment are more likely to use negative coping styles (such as avoidance, self-blame, fantasy, and substance abuse) in adulthood, and less likely to use positive coping styles (such as problem-solving, seeking social support, and positive reappraisal) (45, 46). Conservation of Resources Theory (26, 47) holds that psychological maltreatment is an experience of continuous resource loss. It consumes children’s psychological energy (such as self-esteem, sense of control, and sense of security), making it difficult for them to develop sufficient internal resources (such as self-efficacy and optimism) to effectively cope with subsequent pressures and challenges. Positive coping styles themselves require the input of cognitive, emotional, and behavioral resources, which are lacking in maltreated children. Therefore, low self-esteem and low self-efficacy caused by psychological maltreatment weaken individuals’ confidence in their ability to cope with challenges effectively, thus avoiding positive coping. At the same time, psychological maltreatment impairs children’s cognitive development, which may lead to negative self-schemas (“I am incompetent,” “I am unworthy of being loved”) and world schemas (“The world is dangerous,” “Others are unreliable”). This negative cognitive framework makes it difficult for them to take the initiative to adopt positive strategies to solve problems or seek support (because of the expectation of failure or rejection) (48). In addition, long-term emotional neglect or devaluation hinders the development of emotional regulation ability, making it difficult for individuals to remain calm and think rationally when facing pressure, and instead adopt more primitive and negative coping styles (such as emotional venting or withdrawal).

Stress and Coping Theory (27) emphasizes that stress experiences (such as loneliness) are the result of the interaction between individuals’ evaluation of their relationship with the environment and their coping strategies. Positive coping styles (such as problem-solving, seeking social support, and positive reappraisal) can effectively change individuals’ evaluation of stressors (such as social difficulties and adaptation problems) (reducing their threat), and directly manage stressors or accompanying emotions, thereby reducing the occurrence and persistence of negative emotions (such as loneliness). College students face multiple pressures such as academic studies, interpersonal relationships, and career planning. Adopting positive coping styles (such as actively solving problems, seeking help from friends or teachers, and viewing setbacks with an optimistic attitude) can more effectively deal with specific situations that cause loneliness (such as interpersonal conflicts and adapting to new environments). More importantly, positive coping styles (especially seeking support) themselves promote social connection, meet people’s basic need for belonging, and directly counteract the core experience of loneliness. Cognitive strategies such as positive reappraisal help change the interpretation of social situations (such as regarding a rejection as accidental rather than one’s own defect), thereby reducing negative emotions. The sense of control and efficacy brought by the successful use of positive coping styles can also enhance individuals’ confidence and willingness to actively participate in social interactions.

To sum up, experiences of childhood psychological maltreatment impair individuals’ ability and tendency to develop positive coping strategies. When these college students who have been maltreated enter an environment full of new challenges (establishing new relationships, living independently, academic pressure), due to the lack of effective positive coping resources (such as difficulty in actively seeking support, inability to solve problems effectively, and habitual negative cognition), they find it more difficult to adapt to the environment, establish and maintain satisfactory interpersonal relationships, and thus are more likely to experience profound loneliness.

### 4.4 Research limitations and prospects

Although this study has made some progress in revealing the psychological mechanism between childhood psychological maltreatment and college students’ loneliness, there are still the following limitations. Future research can be improved and deepened from the following aspects: First, the cross-sectional design fundamentally limits causal inference. Although the structural equation model supports the statistical plausibility and theoretical rationale of the proposed dual-path mediation, the observed relationships are correlational. The paths from childhood maltreatment to the mediators and to loneliness, while consistent with theoretical models (e.g., attachment theory), cannot be interpreted as evidence of causal direction or temporal precedence. This is a critical limitation for mediation hypotheses, which are inherently causal in nature. Future research must employ longitudinal or experimental designs to robustly examine the proposed causal pathways and the long-term impact of psychological maltreatment. Second, all variables are measured by self-reported scales, which may have recall bias (such as misreporting or underreporting of childhood maltreatment experiences) and social desirability bias. In the future, multi-source data (such as reports from parents, peers, or teachers) or objective indicators (such as observation of social behavior, physiological indicators such as cortisol levels) can be combined for cross-validation. Third, the samples are concentrated in two universities in Liaoning and Shanghai, mainly including art and engineering students, which may limit the extrapolation of the results. In the future, it is necessary to expand the sample scope (such as students from different regions and cultural backgrounds) and include clinically high-risk groups (such as patients with depression or social anxiety) to improve ecological validity.

## 5 Conclusion

Through a large-sample structural equation model, this study systematically reveals the dual paths of childhood psychological maltreatment on college students’ loneliness: on the one hand, it triggers social withdrawal through “distrust of others,” and on the other hand, it reduces problem-solving efficacy by “weakening positive coping.” The research results deepen the understanding of the mechanisms linking early trauma to later loneliness. Based on the specific magnitudes of the indirect effects observed (12.07% via trust and 16.19% via positive coping), the findings offer empirical support for integrated intervention approaches that simultaneously aim to rebuild interpersonal trust and enhance adaptive coping skills among college students with histories of psychological maltreatment. While the broad concept of combining trust-building and skill-training is not novel in therapeutic practice, this study provides a mechanistic rationale—grounded in a dual-path model—for intentionally targeting these two domains to mitigate loneliness. Future intervention studies should empirically test the efficacy of such integrated programs derived from this model.

## Data Availability

The data during the current study is available in a data repository (doi: 10.17632/dhtnmvdd2k.1).
